# Using the multi-omics approach to reveal the silk composition in *Plectrocnemia conspersa*

**DOI:** 10.3389/fmolb.2022.945239

**Published:** 2022-08-11

**Authors:** Lenka Rouhová, Hana Sehadová, Lucie Pauchová, Miluše Hradilová, Martina Žurovcová, Michal Šerý, Michal Rindoš, Michal Žurovec

**Affiliations:** ^1^ Biology Centre of the Czech Academy of Sciences, Institute of Entomology, Ceske Budejovice, Czechia; ^2^ Faculty of Science, University of South Bohemia, Ceske Budejovice, Czechia; ^3^ Institute of Molecular Genetics, Academy of Sciences of the Czech Republic, Praha, Czechia; ^4^ Faculty of Forestry and Wood Sciences, Czech University of Life Sciences Prague, Prague, Czechia

**Keywords:** fibroin, zonadhesin, mucin, caddisfly, Trichoptera, adhesion, fibers, biomaterials

## Abstract

Similar to Lepidoptera, the larvae of Trichoptera are also capable of producing silk. *Plectrocnemia conspersa*, a predatory species belonging to the suborder Annulipalpia, builds massive silken retreats with preycapturing nets. In this study, we describe the silk glands of *P. conspersa* and use the multi-omics methods to obtain a complete picture of the fiber composition. A combination of silk gland-specific transcriptome and proteomic analyses of the spun-out fibers yielded 27 significant candidates whose full-length sequences and gene structures were retrieved from the publicly available genome database. About one-third of the candidates were completely novel proteins for which there are no described homologs, including a group of five pseudofibroins, proteins with a composition similar to fibroin heavy chain. The rest were homologs of lepidopteran silk proteins, although some had a larger number of paralogs. On the other hand, *P. conspersa* fibers lacked some proteins that are regular components in moth silk. In summary, the multi-omics approach provides an opportunity to compare the overall composition of silk with other insect species. A sufficient number of such studies will make it possible to distinguish between the basic components of all silks and the proteins that represent the adaptation of the fibers for specific purposes or environments.

## 1 Introduction

The larvae of many insect species produce secretory protein fibers and adhesives with remarkable mechanical properties. The best known of these secretory products is silk, produced by lepidopteran and trichopteran larvae for the construction of cocoons in terrestrial environments and of shelters and nets in aquatic environments, respectively. Silk spinning developed in the ancestors of Lepidoptera and Trichoptera about 300 million years ago (Thomas et al., 2020).

The silks of both groups are similar in that they consist of a fibrous core and a sticky coating. The axial fiber of lepidopteran silk is composed of heavy and light fibroin chains in addition to fibrohexamerin; these proteins are produced in the posterior part of the silk glands (SGs). The heavy chain fibroin, in particular, is a crucial component of silk, as its crystal-forming β-sheet regions are responsible for the tensile strength of the fiber (Hayashi et al., 1999). The coating is added in the middle parts of the SGs and usually consists of several sticky proteins called sericins and antimicrobial peptides called seroins. Recent research using “omics” methods has shown that the structure of silk is more complex than previously thought and that its composition needs to be revised. More than 280 proteins were found in the silk of *Bombyx mori* (Zhang et al., 2015), for example, while a similar number have been discovered in the silks of other lepidopteran species studied (Rindos et al., 2021; Rouhova et al., 2021; Volenikova et al., 2022).

The protein composition of silk and the sequences of most trichopteran silk genes are largely unknown. Analysis of silk gland-specific cDNAs from larvae of *Hydropsyche angustipennis*, *Limnephilus decipiens*, and *Rhyacophila obliterata* has confirmed the relatively similar structures of the fibrous core of caddisflies and moths, which consists of two fibroin subunits (Yonemura et al., 2006, Yonemura et al., 2009). Additional partial or even complete fibroin sequences have recently been discovered in other trichopteran species, such as *Hesperophylax* sp., *Parapsyche elsis*, *Stenopsyche tienmushanensis*, and *Stenopsyche marmorata* (Wang et al., 2010; Ashton et al., 2013; Luo et al., 2018; Frandsen et al., 2019). The identification of other silk components requires protein analysis.

A 37 kDa secretory protein, nest-forming protein 1 (Nsfp1), has been found in the silk gland lumen of *Hydropsyche* sp. This may be an example of trichopteran adhesives that are largely different from the serine-rich adhesives known in moths (Eum et al., 2005). Two novel non-fibroin proteins (167 and 132 amino acids long) were identified in *S. marmorata* silk (Bai et al., 2015) and three in *Hesperophylax occidentalis* silk, including peroxinectin, superoxide dismutase 3, and a novel structural component with sequence similarity to the elastic proline, glutamate, valine and lysine-rich (PEVK) region of the mammalian muscle protein titin (Wang et al., 2014). Considering that the number of proteins present in lepidopteran silks reaches the low hundreds, there appear to be numerous trichopteran silk proteins that remain undiscovered.

In this study, we provide a detailed silk analysis of *Plectrocnemia conspersa*, a caddisfly of the suborder Annulipalpia. Its larvae are predatory and build underwater webs to capture their prey. We performed next-generation sequencing (NGS) of the SG-specific cDNA library to obtain the transcriptome by *de novo* assembly. We also collected silk material, which we trypsinized and subjected to peptide mass fingerprinting. We then identified the peptides discovered by matching them to a protein database derived from our RNA-Seq data. Finally, we used the available genomic sequences (Heckenhauer et al., 2019) to infer the repetitive sequences and structures of several full-length silk genes.

## 2 Materials and methods

### 2.1 Biological material

Last instar larvae of *P. conspersa* (Curtis, 1834) were collected in a stream about 7 km east of České Budějovice, in the Czechia (48°59′23.3″N, 14°33′55.3″E). Their species was verified by cytochrome c oxidase I (COI) barcode (Supplementary Figure S1) (Wilson, 2012). The larvae were either used immediately for dissection, fixed for histology, or left in glass containers with aeration to spin new silk fibers, which they do naturally when moved from their original retreat to new environment. This pure silk was collected and used for proteomic analysis and imaging. If the silk was designated for SEM, aluminum holders were placed in the container with the larva. When the larva was removed after spinning, water was gradually removed from the container using a pipette, causing the floating fibers to sink onto the aluminum holder. For proteomics, it was sufficient to pull the fibers out with tweezers.

### 2.2 Scanning electron microscopy and histology

#### 2.2.1 Ultrastructure of silk

Silk fibers spun by caddisflies on the surface of aluminum holders were coated with gold and analyzed using a Jeol JSM-7401F scanning electron microscopy (SEM) (Jeol, Akishima, Japan).

#### 2.2.2 Paraplastic sections

The cuticle of the *P. conspersa* larvae was pierced under the saturated picric acid-based fixative with 3.6% formaldehyde and 2.3% copper acetate supplemented with mercuric chloride (Bouin-Hollande solution) (Levine et al., 1995). Following 1 h of fixation, the larvae were cut into three pieces and then fixed overnight at 4°C. After coarse washing in 70% ethanol, the tissue was dehydrated by an ethanol series (70%, 96%, and 100%, each twice for 20 min) and 100% chloroform (twice for 20 min), embedded in paraplast, and then sectioned to 10 μm.

For the labeling procedure, samples were deparaffinized in 100% xylene (twice for 10 min), rehydrated by subsequent incubation in 96% and 70% ethanol for 5 min each, and washed in distilled water for 5 min. The sections were treated with Lugol’s iodine followed by a 7.5% sodium thiosulfate solution to remove residual heavy metal ions and then washed in distilled water and stained with HT15 Trichrome Stain (Masson) Kit (Sigma-Aldrich, Burlington, United States) according to the manufacturer’s protocol. The stained sections were dehydrated and mounted in DPX mounting medium (Fluka, Buchs, Switzerland). High-resolution images were acquired using a BX63 microscope, DP74 CMOS camera, and cellSens software (Olympus, Tokyo, Japan) and by stitching multiple images together.

### 2.3 Transcriptome preparation

The RNA isolation, purification, and preparation of cDNA libraries were performed as described previously (Rouhova et al., 2021). A MiSeq (Illumina, San Diego, CA, United States) instrument was used to obtain 150-nt long paired-end reads. Two alternative transcriptome assemblies were generated: 1) SeqMan NGen 17.0.2.2 (DNASTAR Lasergene 17), which was used to trim and assemble the raw reads (K-mer size 29), and 2) Galaxy platform (Afgan et al., 2018), which was used for the quality control of the reads (FastQC, Galaxy version 0.72 + galaxy1), trimming (Trimmomatic, Galaxy version 0.38.0, ILLUMINACLIP with default TruSeq2 adapters, SLIDINGWIDOW + MINLEN with default settings), and assembly (Trinity, Galaxy version 2.9.1 + galaxy1, default settings). Both assemblies were checked for completeness with BUSCO (Galaxy version 5.0.0 + galaxy0), using the transcriptome assembly mode (DNA) and the Insecta database. The amino acid sequence database was created using the getorf function (Galaxy version 5.0.0.1, translation from STOP to STOP, minimum 150 nt long ORFs, no flanking nucleotides for output).

### 2.4 Proteomics

The spun silk sample was dissolved in urea, trypsinized, and analyzed by nanoscale liquid chromatography coupled with tandem mass spectrometry (nLC-MS/MS) as previously described (Rouhova et al., 2021). Peptide mass fingerprinting was performed using MaxQuant 1.6.17.0 software (Tyanova et al., 2016). Default settings for false discovery rate (FDR) and minimum peptide length were used (i.e., 1% and seven amino acids, respectively). We also included searches for phosphorylation (phospho ST and STY) and disulfide bonds (Cys-Cys), also provided by MaxQuant.

### 2.5 Database polishing and gene structure construction

Candidate proteins obtained from the proteomic analysis were manually annotated using NCBI BLAST (Altschul et al., 1990), and the presence of signal peptides was predicted using SignalP 5.0 (Almagro Armenteros et al., 2019). The fragmented or misassembled sequences were manually completed and polished against the alternative assembly and the publicly available genome (Heckenhauer et al., 2019) using Local Blast integrated into BioEdit 7.2 (Hall, 1999). Exon-intron boundaries were determined by comparing the cDNA sequences to the genomic one. As a visual aid, we mapped the raw cDNA data to the genome using RNA STAR (Galaxy version 2.7.8a, default settings) and visualized the .bam file in IGV 2.9.4 (Thorvaldsdottir et al., 2013).

### 2.6 qPCR

RNA isolated from four larval tissue types in four biological replicates and three SG regions in three biological replicates was reverse transcribed using the Thermo Scientific RevertAid RT kit (Thermo Fisher Scientific, Vilnius, Lithuania). Subsequently, qPCR reactions were performed in triplicate using the Rotor-Gene Q MDx 2plex HRM instrument (Qiagen, Hilden, Germany). The volume of each reaction was 20 µl and contained 250 nM primer, 4 µl of the mixture HOT FIREPol EvaGreen qPCR Mix Plus (Solis BioDyne, Tartu, Estonia), and cDNA corresponding to 5 ng of the original RNA.

The 2^ΔCt^ calculation with normalization against elongation factor 1-α was used to process the data. Statistical significance was determined using one-way ANOVA and the Tukey HSD test or the Kruskal-Wallis rank-sum test in combination with the Wilcoxon rank sum test where the parametric ANOVA criteria were not met. Primers (Supplementary Table S1) were designed using PrimerSelect 8.0.2 (DNASTAR Lasergene, Madison, United States), and statistical analysis was performed using R 4.0.3 (R Core Team, 2017) in combination with RStudio 1.3.1093 (R Studio Team, 2015).

### 2.7 Phylogenetic analysis

Only coding sequences were used, full length for zonadhesin-like genes and terminal sequences for heavy chain fibroin. MEGA7 software (Kumar et al., 2016) was used to create codon-based alignment with the MUSCLE method. The phylogram was generated using the IQtree server (Nguyen et al., 2015), which includes both the selection of the best substitution model (GTR+F+I+G4 for zonadhesin, TIM2+F+I for FibH5′ and GTR+F+I for FibH3′) (Kalyaanamoorthy et al., 2017) and the generation of the phylogram using the maximum-likelihood algorithm (Bootstrap 1,000x) (Hoang et al., 2018).

## 3 Results

### 3.1 Silk and silk glands ultrastructure

**FIGURE 1:**
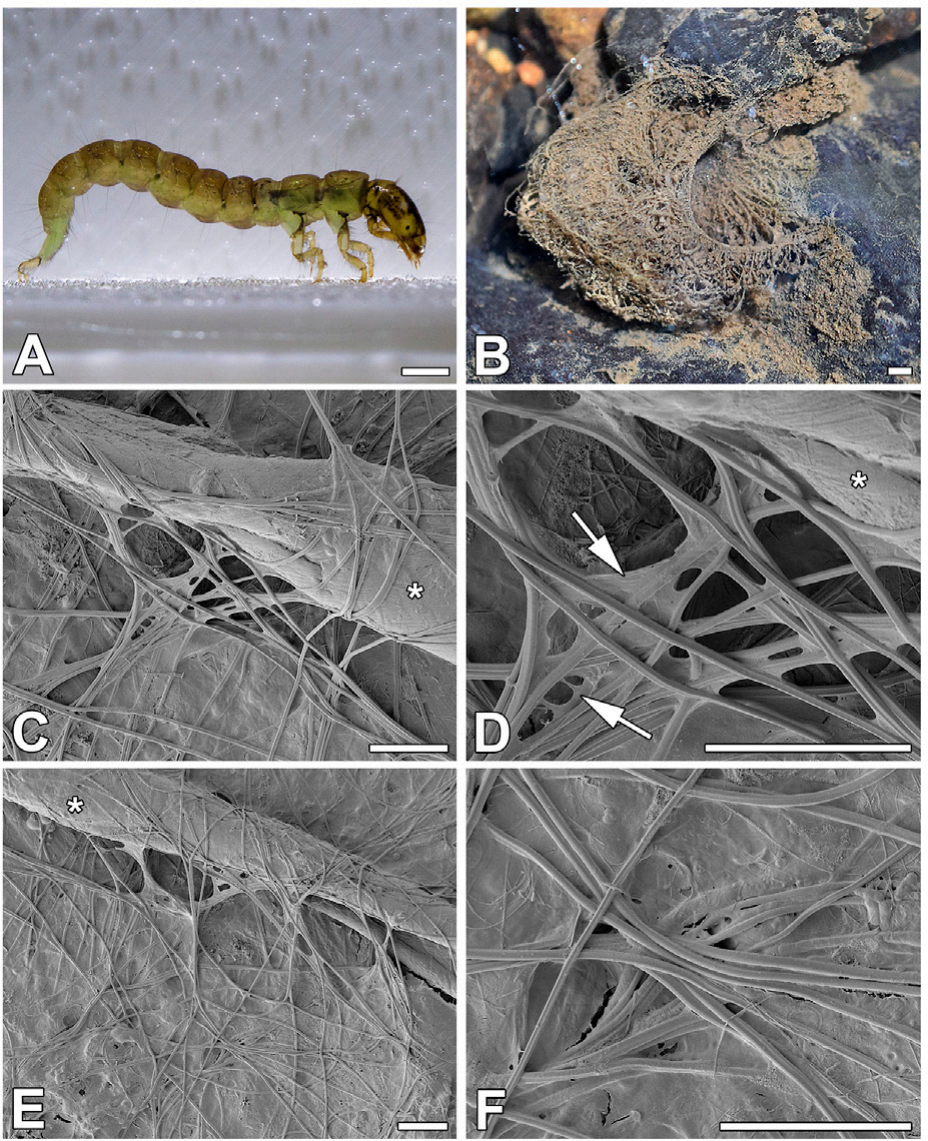
Silk of *P. conspersa*. **(A)** *P. conspersa* larvae; **(B)** *P. conspersa* silken net in the natural environment; **(C–F)** Scanning electron microscopy (SEM) of the silk fibers spun by the larvae in laboratory conditions caught on the surface of an aluminum holder at various magnifications. It is clearly distinguishable that the fibers are composed of two filaments, and they are slightly flattened. The arrows indicate the mass of adhesive material that stretches between the fibers. Asterisks mark a piece of plant debris that the fibres attached to. Scale-bars: **(A,B)** 1 mm; **(C–F)** 10 μm.

The predatory larvae of *P. conspersa* (Figure 1A) do not build portable cases. Instead, they build massive, solid retreats with prey-catching webs (Figure 1B) composed of 0.55 µm (SD = 0.07 µm) thick silk fibers. Since these fibers are produced by paired silk glands, they consist of two interconnected filaments (Figures 1C–F). They are slightly flattened and covered by adhesives that can form membrane-like structures at the crossing points of several fibers (Figure 1D).

**FIGURE 2:**
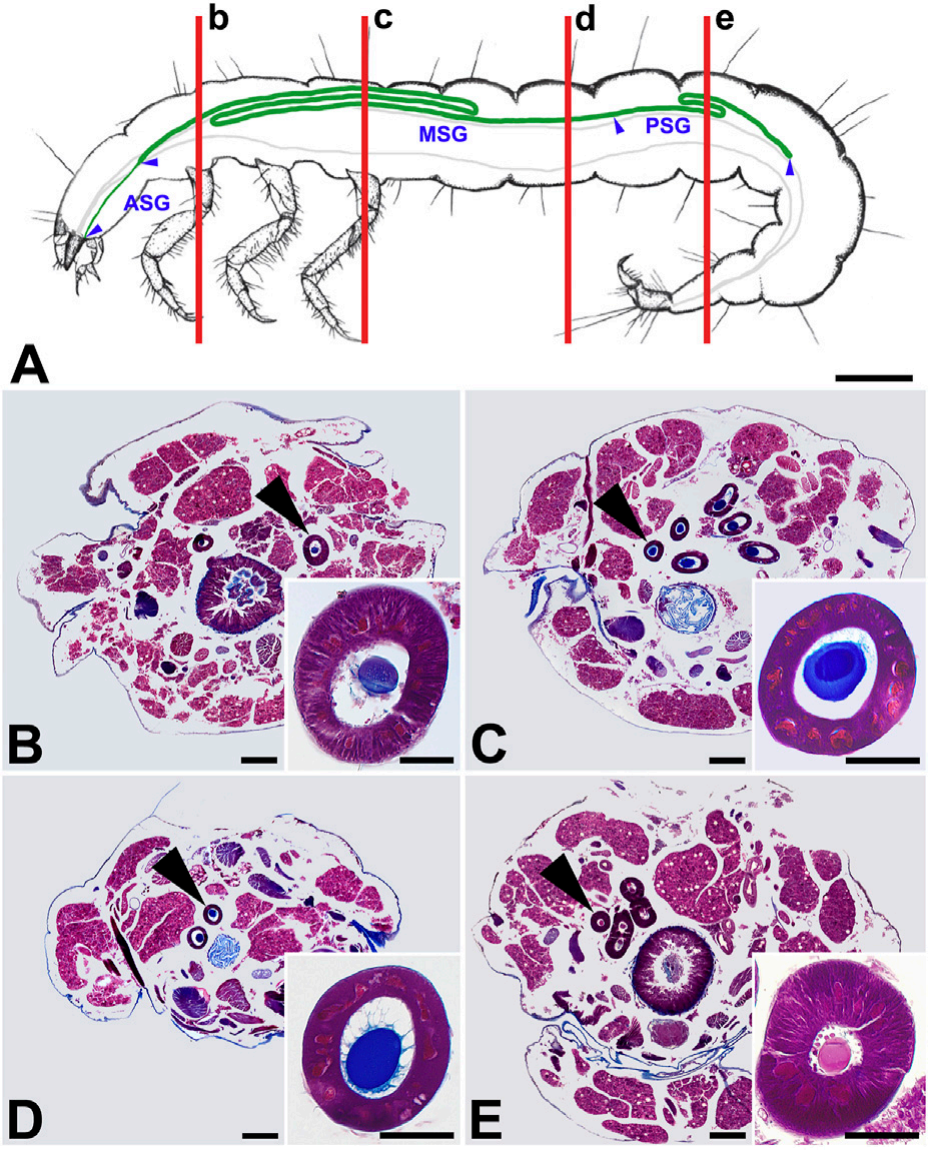
Morphology of the silk gland (SG) from *P. conspersa* last instar larvae. **(A)** Schematic illustration of the SG anatomy in the larval body; blue arrowheads show the boundaries of the SG compartments, i.e., where anterior, middle, and posterior SG (ASG, MSG, and PSG) part. Vertical red lines marked by small letters **(b–e)** refer to the whole-body sections **(B–E)** and show the approximate position where the glands were cut in the respective transverse paraplast sections; **(B–E)** Transverse paraplast sections through the body of a fifth instar larva stained with Masson trichrome stain. Inserted images show higher magnification of the SG section pointed by the arrowheads; **(B–D)** MSG front, middle and rear portions; **(E)** PSG. Scale-bars: **(A)** 1,000 μm; **(B–E)**, 200 μm; inset images, 50 μm.

The SG extends along about three-quarters of the length of the body (Figure 2A), and its cells contain polyploid nuclei along their entire length (Figures 2B–E). Its anterior segment (ASG) is narrow and gradually widens to the middle segment (MSG), which forms a larger Z-shaped loop in the thorax and anterior abdominal segments and another one, smaller, approximately in the middle of the larval abdomen. The diameter of the gland remains the same and decreases slightly only towards the posterior part of the gland (PSG). Otherwise, there is no obvious morphological distinction between the MSG and PSG. However, the smaller second loop is probably part of the PSG, as its sections show that the lumen contains the fibroin core but no envelope proteins (Figure 2E). Other components of the silk are gradually added in the more anterior parts of the SG.

As seen on the paraplastic sections stained with Masson trichrome, the staining of the SG contents changes among the different sections of the gland. While the fibroin is stained red in PSG (Figure 2E), it changes color to blue in MSG (Figures 2B–D). This may be due to a pH change in the SG lumen. Interestingly, the sticky coating has the same color as the fibroin filament.

### 3.2 Candidate silk proteins emerging from the combined -omics analyses

We produced an SG-specific cDNA library from the SGs of *P. conspersa*. Illumina sequencing yielded more than 23 million reads, which were used to generate two alternative assemblies (Table 1).

**TABLE 1:** Comparison of two alternative transcriptome assemblies prepared by Trinity and SeqMan NGen in terms of number and length of contigs and the transcriptome completeness.

	Trinity	SeqMan Ngen
Number of contigs	46,357	12,197
N50 (bp)	3,149	1,685
Length mean (bp)	1,433	1,270
Length median (bp)	587	975
Complete BUSCOs (%)	91.4 (44.6 single copy, 46.8 duplicated)	66.8 (63.8 single copy, 3.0 duplicated)
Fragmented BUSCOs (%)	0.9	7.2
Missing BUSCOs (%)	7.7	26

The Trinity assembly was found to contain more contigs and be more complete (although with more duplicates) than the assembly produced by SeqMan NGen, but the latter’s assembly of repetitive sequences (e.g., fibroin heavy chain) was more efficient (considering the length of the assembled sequence). Therefore, the SeqMan NGen assembly was used as a basis for the primary proteomic analysis. Because the sequences were often fragmented, especially for the repetitive silk proteins, and several close paralogs were incorrectly assembled into fused transcripts, we improved the sequences of the candidate genes by comparing them with the Trinity assembly as well as the publicly available genome (Heckenhauer et al., 2019). As a result, we obtained a sophisticated database of silk protein candidates.

A total of 261 peptides of 67 proteins were detected in the spun-out silk using MaxQuant software and the transcriptome-derived protein sequence database. The proteins are listed in Supplementary Table S2, along with their GenBank accession numbers and structural parameters. Only 23 of the proteins had been predicted to contain a signal peptide for secretion, so these proteins were considered for further analysis. As expected, we detected the fibroin light (FibL) and heavy (FibH) chains but no fibrohexamerin/p25 homolog. In addition, the silk contained a chymotrypsin-like protease (Chtr), endoplasmin (Enpl), serpin (Srp), two mucin-like proteins (Muc1/2), five novel proteins that shared some features with fibroins and were therefore termed “pseudofibroins” (Pfibs), and three additional unannotated proteins, named SGA28, PN20, and LAN32 because of their size and dominant amino acids. The largest category of silk proteins consisted of eight zonadhesin-like proteins (Zons) detected by proteomics, and the zonadhesin family was supplemented with four additional candidates identified on the basis of homology between their transcripts or localized in the same genomic cluster as the other zonadhesins. This thus completed an expanded list of 27 putative silk proteins. The characteristic features of these proteins are listed in Table 2. Strikingly, the vast majority of candidate proteins were hydrophilic.

**TABLE 2:** Candidate silk genes and their properties.

Symbol	GenBank	Genome scaffold	Detection	Exons	Size (kDa)	GRAVY	1st AA (%)	2nd AA (%)	3rd AA (%)
PC-FibL	OL589402	VTFK01000194.1	P + T + G	6	22.951	0.026	A (16.9)	S (12.8)	L (8.2)
PC-FibH	OL589410	Not found	P + T	N/A	Incomplete	−0.462^∗^	S (N/A)	A (N/A)	G (N/A)
PC-Zon1	OL589399	VTFK01000594.1	P + T + G	21	127.505	−0.637	C (14.7)	K (12.3)	G (10.8)
PC-Zon2	OL589400	VTFK01000171.1	P + T + G	16	92.402	−0.363	C (17.4)	G (8.8)	N (8.2)
PC-Zon3	OL589404	VTFK01000594.1	P + T + G	35	196.241	−0.867	C (16.9)	P (12.5)	E (8.6)
PC-Zon4	OL791313	VTFK01000355.1	P + T + G	2	91.619	−0.317	C (16.5)	K (9.3)	P (8.7)
PC-Zon5A	OL791314	VTFK01000355.1	T + G	1	29.038	−0.305	C (15.0)	T (9.9)	K (9.1)
PC-Zon5B	OL791315	VTFK01000355.1	P + T + G	1	28.054	−0.203	C (15.4)	T (9.7)	A (9.4)
PC-Zon5C	OL791316	VTFK01000355.1	P + T + G	1	27.771	−0.241	C (15.4)	T (10.1)	G (9.4)
PC-Zon5D	OL791317	VTFK01000355.1	P + T + G	1	27.826	−0.253	C (16.1)	T (9.7)	G (9.0)
PC-Zon6	OL791321	VTFK01000594.1	P + T + G	12	54.835	−0.498	C (16.4)	P (9.7)	K (8.1)
PC-Zon7	OL405647	VTFK01000355.1	T + G	1	25.605	−0.136	C (14.7)	N,P (10.2)	G (8.2)
PC-Zon8	OL405648	VTFK01000349.1	T + G	10	62.854	−0.287	C (14.8)	P (10.8)	G (7.1)
PC-ZonP	OL405649	VTFK01000355.1	G	1	47.178	−0.529	C (16.7)	P (9.4)	K (8.5)
PC-Pfib1	OL589397	VTFK01000286.1	P + T + G	2	57.335	−0.533	A (32.0)	S (18.3)	T (7.4)
PC-Pfib2A	OL589405	VTFK01000308.1	P + T + G	2	68.014	−0.265	A (33.6)	S (21.3)	G (13.7)
PC-Pfib2B	OL589407	VTFK01000308.1	P + T + G	2	50.612	−0.128	A (40.2)	S (16.9)	K (7.5)
PC-Pfib3	OL589406	VTFK01000061.1	P + T + G	2	43.633	0.114	A (43.6)	S (17.5)	G (7.6)
PC-Pfib4	OL791312	VTFK01000308.1	P + T + G	2	135.347	−0.192	A (32.8)	S (11.7)	G (9.5)
PC-SGA28	OL589401	VTFK01000308.1	P + T + G	3	28.484	−0.876	S (23.1)	G (11.6)	A (9.4)
PC-PN20	OL791318	VTFK01000388.1	P + T + G	2	19.842	−0.785	P (12.4)	N (8.3)	F,S,T,W (6.5)
PC-LAN32	OL791320	VTFK01000028.1	P + T + G	7	32.381	−0.063	L (9.1)	A (8.8)	N (8.4)
PC-Muc1	OL791319	VTFK01000612.1	P + T + G	2	85.053	−0.927	N (29.1)	G (24.4)	A (7.5)
PC-Muc2	OL589398	VTFK01000028.1	P + T + G	19	173.137	−1.248	K (12.7)	E (12.2)	N (8.3)
PC-Chtr	OL589403	VTFK01000093.1	P + T + G	8	30.618	−0.39	G (11.4)	I (7.5)	D (7.1)
PC-Srp	OL589408	VTFK01000117.1	P + T + G	9	43.641	−0.307	K (10.3)	L (9.6)	N (8.8)
PC-Enpl	OL589409	VTFK01000171.1	P + T + G	14	87.328	−0.614	E (9.9)	K (9.0)	D (8.8)

Detection abbreviations: P, protein detected in the silk fibers; T, transcript present in the SG transcriptome; G, sequence found in the genomic database. Besides the exon number and protein size, the grand average of hydropathy (GRAVY) and three most abundant amino acids (AA) with their respective percentages are stated. ^∗^GRAVY was calculated from a partial sequence; actual value may slightly differ.

We further used the proteomic data to search for posttranslational modifications, namely phosphorylation and disulfide bonds. Among our candidate proteins, 19 modified peptides were discovered, 14 with various types of phosphorylation and five with Cys residues involved in disulfide bonds (Table 3). Most of the phosphorylated peptides were from FibH (six) and Muc2 (four). Phosphorylation was also detected in two of the Pfibs. Cys residues involved in disulfide bonds were found mainly in Zons. In addition, two of the peptides that contained cysteines were assigned to Zon8 and ZonP, which were not detected by any unmodified peptide in the initial proteomic analysis.

**TABLE 3:** Posttranslational modifications detected in the silk fibers by the peptide mass spectrometry.

Protein	Modifications	Peptide (Modification localization probability)
PC-FibH	Phospho (STY); Phospho (ST)	AS(0.002)AAAS(0.498)AS(0.5)AEGGWGHGR
PC-FibH	Phospho (STY); Phospho (ST)	ASAAAS(0.5)AS(0.5)AEGGWGR
PC-FibH	Phospho (STY)	ISAKQS(1)VEHVEAAVK
PC-FibH	Phospho (ST)	LLQEDDYIEANS(1)RGELVEK
PC-FibH	Phospho (STY)	LS(0.005)T(0.237)S(0.379)S(0.379)AGHGYVHK
PC-FibH	Phospho (STY)	QS(1)VEHVEAAVK
PC-Muc1	2 Phospho (ST)	VS(1)YAT(1)KIVAGAK
PC-Muc2	Phospho (STY)	LS(0.188)FNS(0.812)EEDDDMLYYINPFENYDK
PC-Muc2	Phospho (ST)	NKNQNANT(0.123)KS(0.877)K
PC-Muc2	Phospho (ST)	NQNANT(0.955)KS(0.045)K
PC-Muc2	Phospho (STY); Phospho (ST)	S(0.003)VS(0.497)IQKNKNQNANT(0.5)K
PC-Pfib1	Phospho (ST)	ANAGGNHS(0.995)VES(0.005)INIR
PC-Pfib1	2 Cys-Cys	TAC(1)AGAGC(1)VHYR
PC-Pfib4	Phospho (ST)	IPNSNT(0.5)S(0.5)PEVR
PC-Pfib4	Phospho (STY)	S(1)GGGGGGLYGPNAGCGGR
PC-Zon2	Cys-Cys	KNEVLDLC(0.067)YYSC(0.928)PPQTC(0.004)DAIGK
PC-Zon3	2 Cys-Cys	C(1)PNEVYVINSDLGEPTC(1)TDPNPK
PC-Zon8	2 Cys-Cys	DC(1)PPRLVC(1)IK
PC-ZonP	Cys-Cys	EPKLC(0.454)EYC(0.274)KPSEC(0.272)K

Presence of the modification in the peptide was evaluated by false discovery rate (threshold 1%). Some of the modified peptides contained multiple target amino acids. Thus, the localization probabilities, calculated by the MaxQuant software, are stated in parentheses.

### 3.3 Characterization of silk genes

**FIGURE 3:**
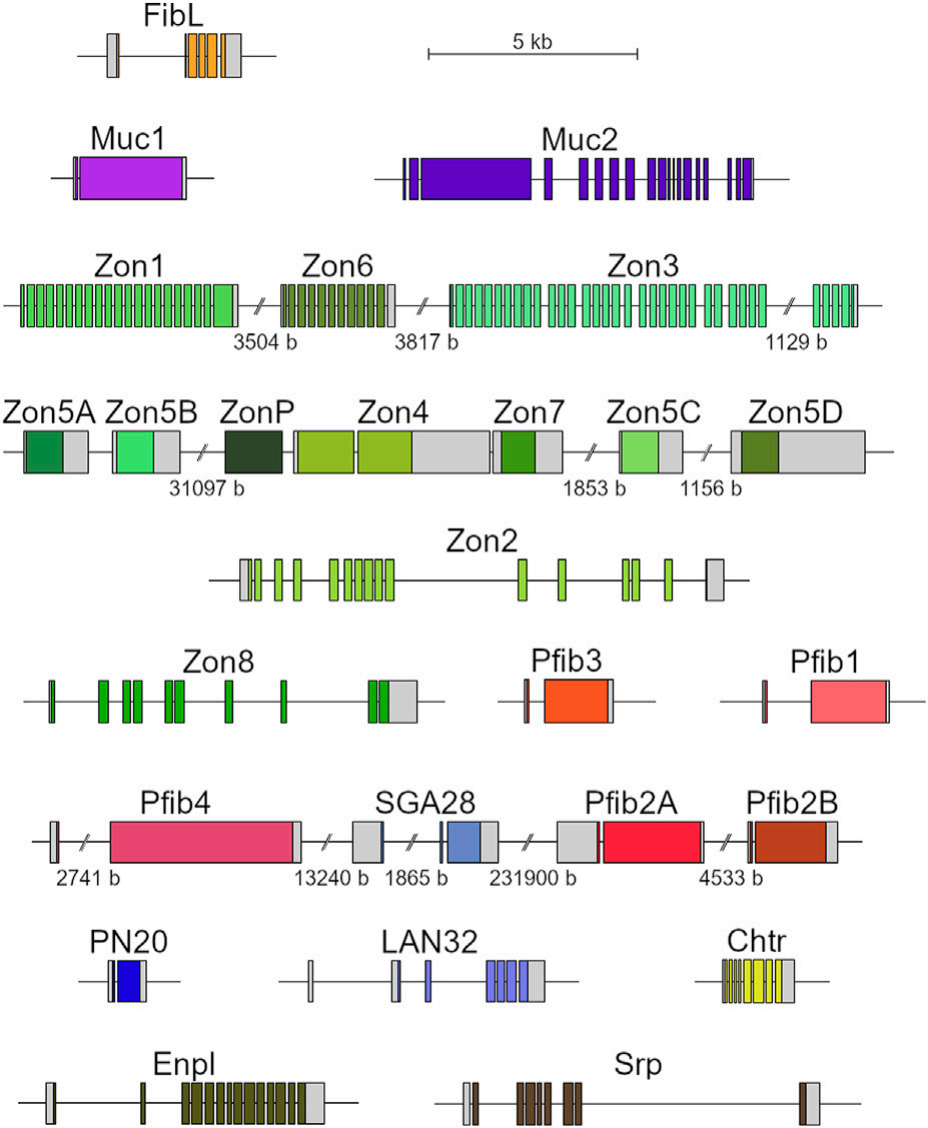
Diagrams of genes encoding silk proteins. Untranslated regions (UTRs) in grey; coding sequences in color (similar hues represent genes encoding proteins of a similar category); genes localized at the same genomic scaffold depicted in clusters; scale breaks in the intergenic regions labelled with the region lengths.

As part of the polishing and completion of the candidate genes, we used the existing genomic sequences (Heckenhauer et al., 2019) to establish the full-length repetitive sequences and gene structures. Unfortunately, the FibH gene was not found in this genomic database, but all the other candidate genes were located, and their exon-intron diagrams were generated (Figure 3).

Analysis of the genomic sequences showed some of the silk genes to be arranged in clusters. This was particularly striking in the genes encoding Zons. In addition, there appeared to be two types of Zons: monolithic, which are usually intronless (Zon5A-D, Zon7, ZonP) or have only one intron (Zon4), and modular, which consist of numerous short, repeating exons and introns (Zon1-3, Zon6, Zon8). Zon genes tend to cluster with the same types, indicating the possibility of frequent gene duplications. Maximum likelihood analysis also clustered these genes together, supporting this hypothesis (Supplementary Figure S2). The Zon5A-D genes are nearly identical (92.8% nucleotide identity between the two most distant genes, Zon5B and ZonD), suggesting that they arose here from a recent duplication.

Two of the identified pseudofibroin genes, Pfib2A and Pfib2B, were also adjacent and had very similar sequence (59.2% identity at the nucleotide level, 54.6% amino acid identity, 61.2% similarity). A common feature of all Pfib genes is the presence of two exons, the first of which is very short and encodes only part of the signal peptide. In addition, Pfib protein sequences share the motifs Ser-Ala and Ser-Gly and some degree of repeatability, which is also true for FibH (Table 4). However, the Ser-Ala and Ser-Gly motifs are shorter, and the repeats are less regular than FibH. While FibH and Pfib4 both contain periodic repeats that include both of these motifs, other Pfibs tend to group the Ser-Ala and Ser-Gly motifs separately, forming distinct N- and C-terminal domains (especially Pfib2A and Pfib2B). In addition, Pfibs contain a higher proportion of charged amino acids (both positive and negative) and more cysteine residues (Table 4).

**TABLE 4:** Comparison of fibroin heavy chain and pseudofibroins.

Protein	Ser-Ala motifs		Ser-Gly motifs		Charged amino acids		Cysteines	
	Length	Localization	Length	Localization	Abund.	Localization	Abund.	Localization
FibH	+++	periodically repeating	++	periodically repeating	+	periodically repeating	+	C-terminus
Pfib1	+	irregular	+/++	shorter N-terminal domain	+++	basic N-terminal domain, mixed C-terminal domain	+	both termini, between N-terminal and C-terminal domain
Pfib2A	+	larger N-terminal domain	+/++	larger C-terminal domain	++	mixed N-terminal domain, uncharged C-terminal domain	++	N-terminus, C-terminal domain
Pfib2B	+	larger N-terminal domain	++	shorter C-terminal domain	++	mixed N-terminal domain, uncharged C-terminal domain	+++	randomly scattered
Pfib3	++	irregular, rather N-terminal	+	irregular, rather C-terminal	+	randomly scattered, more near the N-terminus	+++	randomly scattered
Pfib4	+/++	periodically repeating	+	periodically repeating	+++	periodically repeating with acidic and basic clusters	++	both termini

The length and abundance of certain amino acids and motifs are categorized, and localization is further described.

**FIGURE 4:**
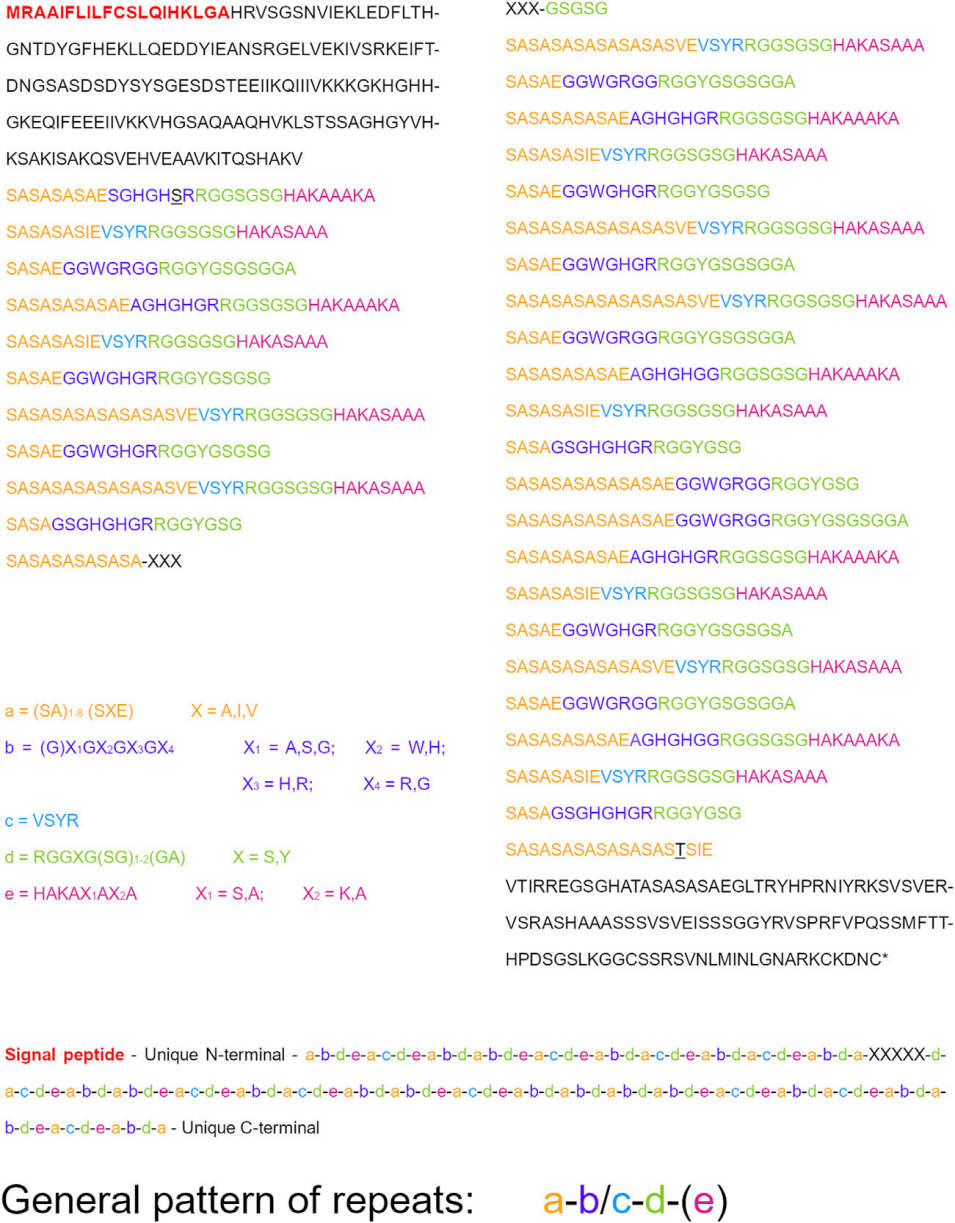
Structure of the repeats in FibH amino acid sequence. Different sections labelled by letters format: red bold—signal peptide; black—terminal unique sequence; yellow—repeat a; purple—repeat b; blue—repeat c; green—repeat d; pink—repeat e; black underlined—consensus-breaking amino acids. The sequences of the repeat sections are simplified to general formulae, where X letters stand for variable amino acids (the options for the particular Xs are always stated next to the formula). Parentheses with a numeric interval label variable number of the amino acids inside them. Parentheses without any numbers label amino acids that are not always present. The order of the individual repeat sections are summarized into a general pattern, where the mutual exclusiveness of repeat sections “b” and “c” is represented by a slash, whereas the repeat section “e” is in parentheses, indicating that it is not always present.

Finally, we analyzed the properties of *P. conspersa* FibH. Although the full-length sequence was not available in the genome database, we found fairly long fragments of the cDNA sequence at both the 5′ and 3′ ends in our SeqMan Ngen assembly. Translation of these fragments provided not only the two conserved ends but also sections of repetitive sequence long enough to infer repeat composition and organization (Figure 4). Basically, there are five types of modules that form the repeat units: a = [SA]_1–8_(SXE); b = (G)XGXGXGX; c = VSYR; d = RGGXG[SG]_1–2_(GA); e = HAKAXAXA (the amino acids in parentheses are not always present). The repeating unit always contains modules “a” and “d” with either module “b” or “c” between them. Module “e” is not present in every repeat; it appears to be absent in approximately half of the units (Figure 4). The overall pattern of repetitions can be simplified thus: a-b/c-d-(e).

### 3.4 Tissue specificity of candidate proteins

**FIGURE 5:**
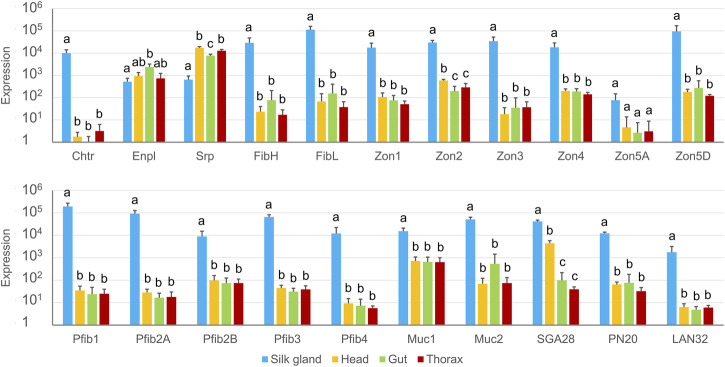
Tissue specificity of candidate genes expression measured by qPCR. The scale is logarithmic; error bars show standard deviation; there were four biological replicates, and statistically significant differences (Kruskal-Wallis + Wilcoxon rank sum test) are represented by different letters.

The transcriptional specificity of the newly predicted genes was determined for 21 of the candidate proteins (six of the total 12 Zons were chosen as representatives); all but two, Enpl and Srp, were found to be SG-specific (Figure 5). Zon5A, which was not originally detected in the proteomic analysis, did not show statistically significant specificity, probably due to its very low expression, but was nevertheless predominantly expressed in SGs.

**FIGURE 6:**
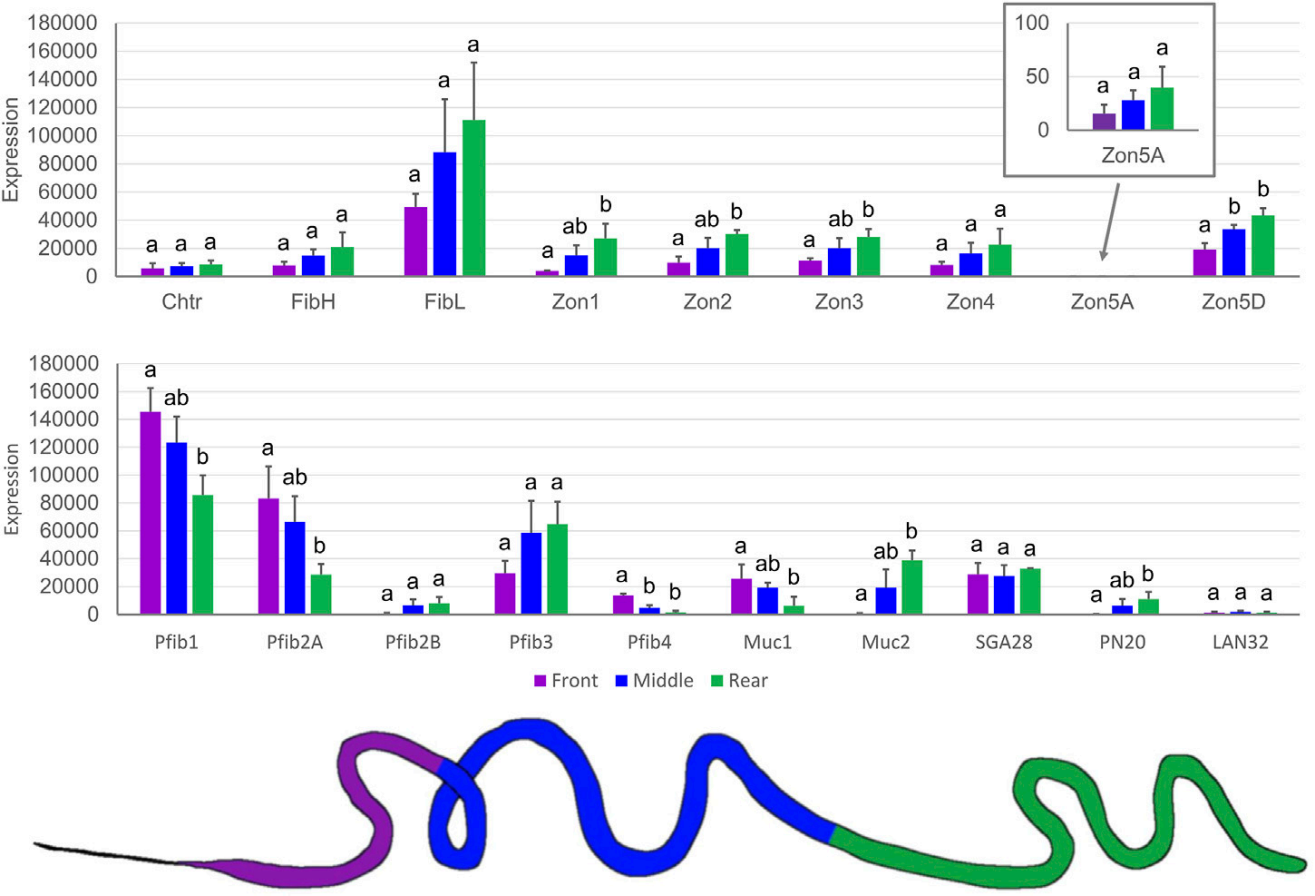
Expression of candidate genes in three parts of the SG measured by qPCR. The color-labeled parts of the SG are shown under the chart. The scale is linear; error bars show standard deviation; there were three biological replicates, and statistically significant differences (One way ANOVA + Tukey HSD test) are represented by different letters.

The tissue specificity of SG-specific genes was further localized within SG compartments. Because there was no clear morphological distinction between the MSG and PSG, we divided SGs into three regions of approximately equal length (Figure 6). This might be the reason why the expression profiles looked like gradients and significant differences were observed mainly between the anterior and posterior parts of the SG (Figure 6). Four proteins predominated in the anterior third of the SG: Pfib1, Pfib2A, Pfib4, and Muc1. Interestingly, Muc2 was predominantly expressed in the posterior (rear) region, similar to PN20 and zonadhesin-like proteins. However, the expression prevalence in the posterior part of SGs sometimes did not reach the statistical significance shown for Zon4, Zon5A, and also for the light and heavy chain fibroins.

## 4 Discussion

In this article, we have characterized the SG and analyzed the silk of caddisfly *P. conspersa* by a combination of SG-specific transcriptome analysis and peptide mass fingerprinting. Full-length sequences of candidate silk genes were extracted from the existing genome database (Heckenhauer et al., 2019), and diagrams of their exon-intron structures were generated. In addition to annotating and describing the silk proteins in silico, we have also verified the specificity of their expression and further investigated their expression pattern in the SG parts.

The study of *P. conspersa* SG allowed us to make comparisons with other known silk components from caddisflies and moths. The *P. conspersa* SGs are longer than the body and folded into Z-shaped loops in two regions. The *P. conspersa* ASG is narrow like in *B. mori*, but, compared to the silkworm, the difference between MSG and PSG is morphologically hardly noticeable. However, at the transition between these two SG compartments, there is a change in the fibroin filament staining in the lumen of the SG. A similar effect was also observed in the SGs of *Yponomeuta cagnagella* (Volenikova et al., 2022), where the Masson trichrome staining of the FibH anteriorly changed from blue to red. These staining differences may be explained by pH changes in the SG lumen, which were previously observed in lepidopteran SGs (Miyake and Azuma, 2008). Interestingly, the coating proteins deposited on the fiber in the MSG were stained the same color as the fibroin core. This is in contrast to moths, where the staining of sericins tends to contrast with fibroins (Rouhova et al., 2021; Volenikova et al., 2022). This suggests that the properties of the coat proteins of *P. conspersa* may be similar to fibroin, whereas the difference between sericins and fibroins of Lepidoptera is much greater.

Proteomic analysis of *P. conspersa* silk revealed 67 proteins, 23 of which were predicted to contain a signal peptide for secretion. Four additional candidates were added based on homology and localization in genomic clusters with related silk genes. The presence of intracellular proteins in silk is a common phenomenon observed in numerous moth species (Zhang et al., 2015; Rindos et al., 2021; Rouhova et al., 2021; Volenikova et al., 2022). It is likely that the mechanism of secretion in SGs of Lepidoptera and Trichoptera is similar to the apocrine secretion known from the salivary glands of *Drosophila melanogaster* (Farkaš et al., 2014). Compared to moths, the number of proteins in *P. conspersa* silk was remarkably low. For example, the respective numbers of total and secretory proteins were 120 and 79 in *Y. cagnagella* (Volenikova et al., 2022), 136 and 59 in *Pseudoips prasinana* (Rindos et al., 2021), and 202 and 101 in *Tineola bisselliella* (Rouhova et al., 2021). This difference in the number of detected proteins can be explained by the aquatic environment, in which the numerous soluble non-structural proteins that are not abundant, such as housekeeping contaminants, can be easily washed away.

The silk of *P. conspersa* differs from that of the land spinners in the quality as well as quantity of constituents. While both FibH and FibL proteins were detected, no protein resembling lepidopteran fibrohexamerin was identified. To our knowledge, no other study of trichopteran silk has detected a homolog of this protein, which appears to be unique to moths. Unsurprisingly, we found no apparent homolog of Lepidoptera seroins. Because the silk of *P*. *conspersa* is used in the form of a temporary prey-catching net, the presence of bacteria is probably more tolerable than in cocoons that protect metamorphosing individuals, and degradation caused by microbial enzymes may be prevented by other means (see below). The presence of sericins is questionable, as they are so diverse that identification by homology based on moth data is nearly impossible.

The basic constituents of Lepidoptera and Trichoptera silk are fibroins. The light chain of fibroin is highly conserved, with 50% amino acid identity and 65% similarity to *S. marmorata* FibL (Bai et al., 2015), the closest known ortholog of *P. conspersa* FibL. Previously performed phylogenetic analysis (Heckenhauer et al., 2019) grouped *P. conspersa* FibL with other FibL proteins of the caddisfly suborder Annulipalpia. The gene structure of FibL contains six exons, like its orthologs in *H. angustipennis* and *R. obliterata* (Yonemura et al., 2009). Unfortunately, the complete gene structure of FibH could not be examined here due to the incompleteness of the genome database (Heckenhauer et al., 2019). However, we obtained fairly long sequence fragments from both ends of the transcript, which allowed us to describe the motifs and infer the organization of the repeats (Figure 4, *Results* section).

The (SX)_n_ motifs described in a variety of Trichoptera species (Yonemura et al., 2006, 2009; Wang et al., 2010; Ashton et al., 2013), were also detected in *P. conspersa* FibH. Unlike the other Trichoptera species, where X represented mostly nonpolar but also other types of amino acids, the (SX)_n_ motifs in *P. conspersa* mostly contained Ala residues except for the last X in the stretch, which often stood for Val or Ile. On the other hand, the length of these sections seemed to be more variable in *P. conspersa*; the other species mostly had four SX doublets per section. As in the other caddisflies, the (SX)_n_ motifs in *P. conspersa* were usually followed by Glu. We also observed the GGX motifs (repeats “b” and “d”) known from other species, but they did not form longer stretches, such as in *R. obliterata* (Yonemura et al., 2009) and the family Limnephilidae (Yonemura et al., 2006; Ashton et al., 2013). Instead, they were more scattered, similar to *S. marmorata* (Wang et al., 2010) and *H. angustipennis* (Yonemura et al., 2006). However, we did not observe any GPXGX motif that was previously known from all other FibH repeats in Trichoptera (Yonemura et al., 2006, 2009; Wang et al., 2010; Ashton et al., 2013). Indeed, there is no Pro residue in the known repetitive region of *P. conspersa* FibH. In contrast, Ala, the second most abundant amino acid, is highly overrepresented in *P. conspersa* compared to other caddisflies (Yonemura et al., 2006, 2009; Wang et al., 2010; Ashton et al., 2013), making *P. conspersa* FibH more similar to that of Lepidoptera.

Another feature that links *P. conspersa* FibH to the heavy chain fibroins of Lepidoptera rather than to its orthologs of Trichoptera is the presence of three Cys residues at the C terminus (Z̆urovec and Sehnal, 2002; Rindos et al., 2021; Rouhova et al., 2021). To date, only two have generally been observed in Trichoptera (Yonemura et al., 2006, 2009; Wang et al., 2010; Ashton et al., 2013). Interestingly, the clustering of *P. conspersa* FibH (both 5′ and 3′ end) with its trichopteran orthologs was not supported by bootstrapping in the maximum likelihood analysis, so its position between the well-defined trichopteran and lepidopteran clusters could not be clarified (Supplementary Figure S3). While Trichoptera silks tend to have fewer crystalline regions due to their excess of charged and bulky residues, Lepidoptera silks with Gly- and Ala-rich FibH can form a high proportion of crystalline structures (Engster, 1976). Interestingly, the crystalline regions of FibH proteins in pyralid moths contain not only Gly and Ala but also a considerable amount of Ser (Fedic et al., 2003). Thus, the Ser-Ala alternations in *P. conspersa* FibH might also represent crystal-forming domains. As in moths, *P. conspersa* fibroins tend to be expressed in the posterior part of SGs.

In addition to fibroins, there are several other proteins found in both moths and caddisflies. Zons have been commonly found in silk throughout the order Lepidoptera (Zhang et al., 2015; Zurovec et al., 2016; Kludkiewicz et al., 2019; Rindos et al., 2021; Rouhova et al., 2021), and homologous transcripts have also been detected in spider silk glands (Berger et al., 2021). However, our previous study in *T. bisselliella* showed that they are mainly expressed in the MSG (Rouhova et al., 2021), whereas in *P. conspersa*, we detected their transcripts in the posterior part of SGs. Moreover, the number of Zons in *P. conspersa* is greater than in the Lepidoptera whose silks were studied. The duplications and losses of Zons are probably very frequent since we found four almost identical Zons in *P. conspersa,* and the maximum likelihood analysis revealed no orthology with the Zons of *T. bisselliella* (Supplementary Figure S2). The exceptionally high content of Cys residues and their epidermal growth factor (EGF) domain-like patterns make Zons similar to trypsin inhibitor-like (TIL)-type protease inhibitors that suppress microbial proteases (Li et al., 2015). If *P. conspersa* Zons have such a function, their duplication could be important for fiber durability. Larger proteins with the potential for Cys-Cys cross-linking appear to be a more effective option for protection against degradation than the elimination of microbes by antimicrobial peptides, which are small and may be difficult to retain in fibers in the aquatic environment.

Another regular component of lepidopteran silks that we observed in *P. conspersa* are mucins (Zhang et al., 2015; Kludkiewicz et al., 2019; Rouhova et al., 2021), suggesting that they may have a conserved function in silks. Two proteins identified in *P. conspersa* silk were assigned to this category. Muc1 contains a CXCXC motif near the C-terminus, which is a common feature with the Muc1 of *T. bisselliella* (GenBank MW244683, Rouhova et al., 2021) and *Y. cagnagella* (GenBank MZ981775, Volenikova et al., 2022). Muc2 was annotated based on structure prediction by the Phyre2 tool (Kelley et al., 2015), which used human Muc2 as the closest reference. Interestingly, Muc1 tends to be expressed in the anterior part of the SG, whereas Muc2 predominates in the PSG. Finally, serine proteases have also been repeatedly detected in moth silk, and—as in *P. conspersa*—their expression also proved to be SG specific (Rouhova et al., 2021; Volenikova et al., 2022). However, their function in silk remains to be elucidated.

On the other hand, we found several new *P. conspersa* proteins that have no obvious homologs in moths. The most striking group were the five proteins called Pfibs. Their expression was observed to increase mainly in the anterior part of the SG (Pfib1, Pfib2A, and Pfib4), suggesting that they probably coat the fibroin core. However, none of them seem to resemble the sericins of Lepidoptera. Instead, the Pfibs strikingly resemble FibH, although they are shorter. This could explain the similarity in the coloration of the fibroin core and its sheath. Unlike lepidopteran sericins, which are generally much more hydrophilic than FibH (Zurovec et al., 2016; Kludkiewicz et al., 2019), the hydrophilicity of Pfibs is comparable to or even lower than that of FibH due to their similar amino acid composition, including Ser-Ala- and Ser-Gly-rich motifs (Table 4). Moreover, Pfib genes have two exons: a short 5′-terminal encoding only part of the signal sequence and a long repetitive 3′-terminal encoding the rest of the molecule. This arrangement is typical of FibH genes in moths (Sezutsu and Yukuhiro, 2000; Sezutsu and Yukuhiro, 2014; Zhou, 2000; Z̆urovec and Sehnal, 2002) and has also been observed in two Trichoptera species (Yonemura et al., 2009).

**FIGURE 7:**
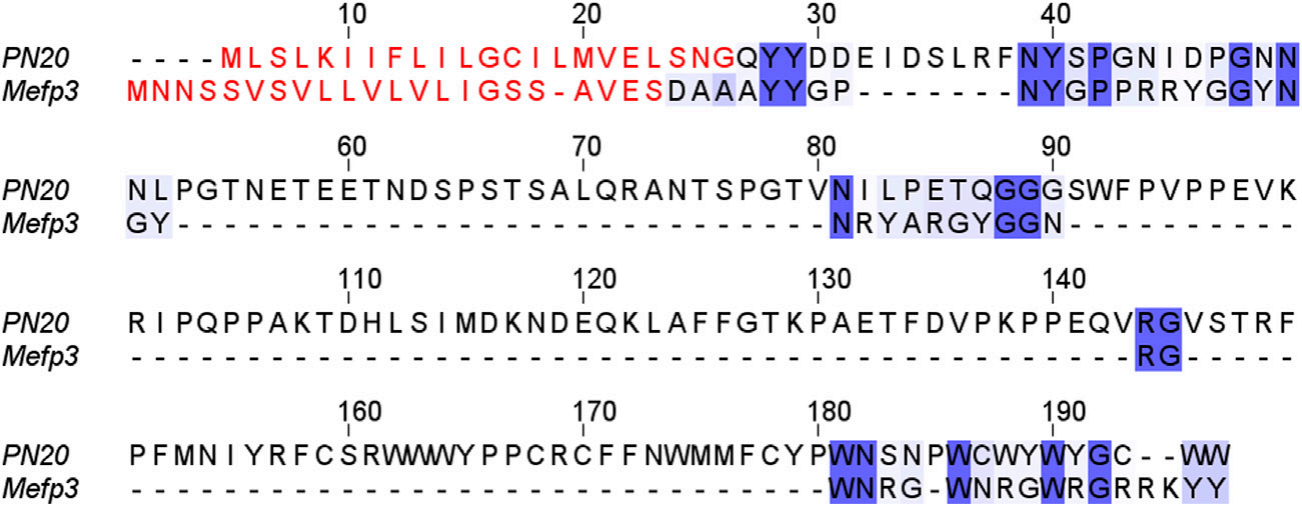
Comparison of *P. conspersa* PN20 and *Mytilus edulis* Mefp3. Signal peptides labelled with red letters; identical/similar amino acids highlighted by shades of blue. Noticeably, a substantial proportion of the identities/similarities is represented by aromatic amino acids.

In addition to Pfibs, SGA28, LAN32, and PN20 were also among the new proteins. The latter is of particular interest because it is small and contains a considerable amount of aromatic amino acids. Similar properties have been observed for the mussel adhesion protein Mefp3 (Figure 7), which serves as a primer in adhesion plaques formation (Lee et al., 2006). Wet adhesion described in mollusks is mediated by Tyr residues converted to Dopa (Lee et al., 2006) or by posttranslational modifications of Trp residues (Zhao et al., 2009). Since both Tyr and Trp are abundant in PN20, we can speculate that it is responsible for adhesion to the substrate.

Interestingly, none of the previously described non-fibrous trichopteran silk proteins from other species were detected in *P. conspersa* silk. The transcript of superoxide dismutase 3 detected in *H. occidentalis* silk (Wang et al., 2014) was found among the transcriptomic sequences, but the proteomic analysis did not confirm that the protein was present in the spun-out silk. The other two *H. occidentalis* candidate silk proteins (peroxinectin, PEVK-like) did not even have obvious homologs among the *P. conspersa* SG-specific transcripts. Although *P. conspersa* belongs to the Annulipalipia suborder (Thomas et al., 2020), homologs of other Annulipalpia silk proteins, such as *Hydropsyche* Nsfp1 and *S. marmorata* Smsp2 and Smsp4, were also not detected at the transcript level. This suggests that some trichopteran silk proteins may be highly specific to lower taxa.

In summary, this is the first multi-omics study to have described the silk composition of a caddis fly. It has not only presented a comparison of the protein composition of trichopteran and lepidopteran silks in all their complexity but also introduced several novel silk proteins. Studies of this type provide candidates for future applied research and annotation references for the ever-expanding sequencing projects in this area.

## Data Availability

The datasets presented in this study can be found in online repositories. The names of the repository/repositories and accession number(s) can be found in the article/Supplementary Material.
