# Supplementary material for: Using the multi-omics approach to reveal the silk composition in Plectrocnemia conspersa
Source: Front Mol Biosci. 2022 Aug 11;9:945239. doi: 10.3389/fmolb.2022.945239 (PMC9432349; doi:10.3389/fmolb.2022.945239)
Supplement: Supplementary file 3 [file DataSheet1.docx]

Supplementary Material

# Supplementary Data

## Supplementary Tables captions

**Supplementary table 1.** List of primers used for qPCR.

**Supplementary table 2.** Complete list of proteins detected by proteomics. Includes the GenBank accession number, information about whether or not the signal peptide for secretion is present, and details about the proteomic detection.

## Supplementary Figures


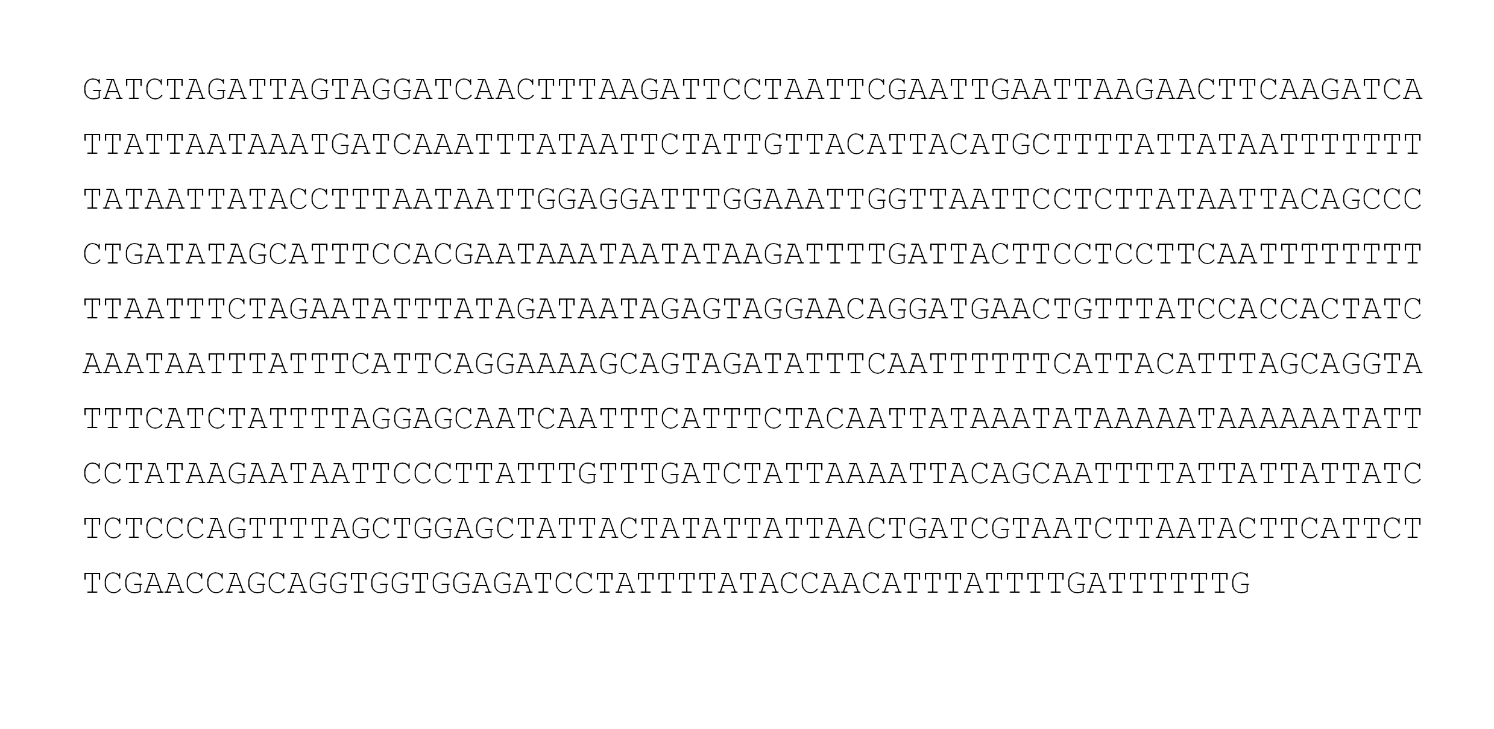


**Supplementary Figure 1.** Partial cytochrome c oxidase I (COI) sequence obtained from *P.conspersa* samples.


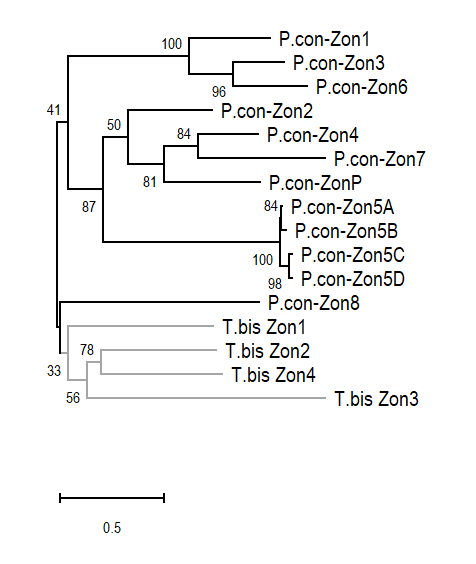


**Supplementary Figure 2.** Maximum likelihood tree of coding sequences of zonadhesin-like genes of *P.conspersa* (P.con). Statistical support (bootstrap values) is shown on the branches. The zonadhesin-like genes of *Tineola bisselliella* (T.bis) were used as outgroups (indicated by grey branches). Their GenBank accession numbers are: Zon1 (MW244684.1), Zon2 (MW244695.1), Zon3 (MW244704.1), and Zon4 (MW244710.1). The analysis showed that the modular *P. conspersa* zonadhesin-like proteins encoded by genes on the same genomic scaffold (Zon1, Zon3, and Zon6) were significantly clustered. The monolithic (single-exon) zonadhesin-like proteins also form a cluster in which Zon5A-D, in particular, are very close to each other. Interestingly, this cluster also included Zon2, which was not part of the same genomic scaffold as the other genes in this cluster. The position of Zon8 is unclear. It tends to cluster more with the outgroup genes, although statistical support is low.


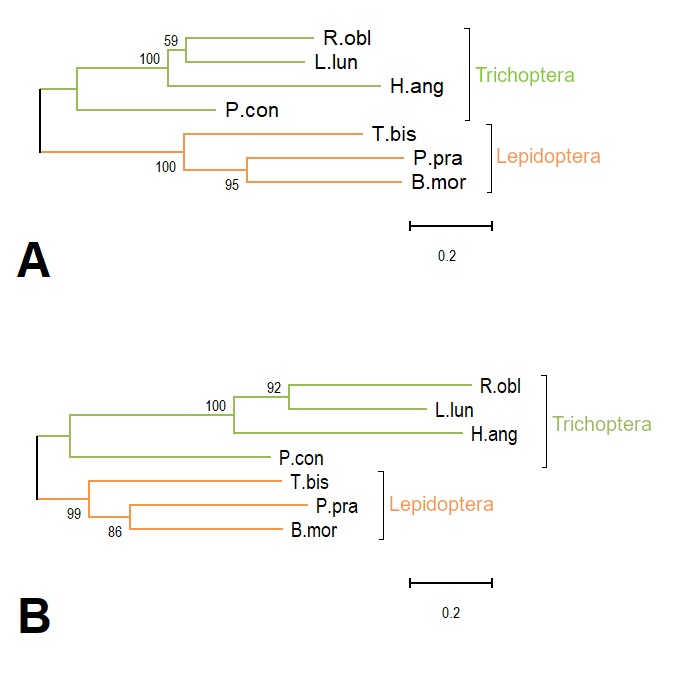


**Supplementary Figure 2.** Maximum likelihood trees of the partial coding sequences of FibH. (A) FibH 5’ end, (B) FibH 3’ end. *P. conspersa* (P.con) FibH was compared with FibH sequences from other caddisflies (green branches) and moths (orange branches). The other species were: *Rhyacophila obliterata* (R.obl, 5’ end - AB354587.1, 3’ end - AB354588.1), *Limnephilus lunatus* (L.lun, FibH sequence from genome database - GCA_917563855.2), *Hydropsyche angustipennis* (H.ang, 5’ end - AB214506.2, 3’ end - AB354592.1), *Tineola bisselliella* (T.bis, MW244680.1), *Pseudoips prasinana* (P.pra, MW373748.1), and Bombyx mori (B.mor, NM_001113262.1). The analysis revealed two clusters, trichopteran and lepidopteran. However, the position of *P. conspersa* FibH in the trichopteran cluster was not statistically supported. It confirms that it differs from most trichopteran heavy chain fibroins and shares some features with the lepidopteran ones, suggesting that it might be ancestral to the other trichopteran FibH.
